# Keeping Your Eyes Continuously on the Ball While Running for Catchable and Uncatchable Fly Balls

**DOI:** 10.1371/journal.pone.0092392

**Published:** 2014-03-26

**Authors:** Dees B. W. Postma, A. Rob den Otter, Frank T. J. M. Zaal

**Affiliations:** Center for Human Movement Sciences, University Medical Center Groningen, Sector F, University of Groningen, Groningen, the Netherlands; University of Texas at San Antonio, United States of America

## Abstract

When faced with a fly ball approaching along the sagittal plane, fielders need information for the control of their running to the interception location. This information could be available in the initial part of the ball trajectory, such that the interception location can be predicted from its initial conditions. Alternatively, such predictive information is not available, and running to the interception location involves continuous visual guidance. The latter type of control would predict that fielders keep looking at the approaching ball for most of its flight, whereas the former type of control would fit with looking at the ball during the early part of the ball's flight; keeping the eyes on the ball during the remainder of its trajectory would not be necessary when the interception location can be inferred from the first part of the ball trajectory. The present contribution studied visual tracking of approaching fly balls. Participants were equipped with a mobile eye tracker. They were confronted with tennis balls approaching from about 20 m, and projected in such a way that some balls were catchable and others were not. In all situations, participants almost exclusively tracked the ball with their gaze until just before the catch or until they indicated that a ball was uncatchable. This continuous tracking of the ball, even when running close to their maximum speeds, suggests that participants employed continuous visual control rather than running to an interception location known from looking at the early part of the ball flight.

## Introduction

Catching a fly ball not only adds to a good result in a baseball game but also keeps fascinating spectators and scientists alike. A particularly famous catch was the one made by Willie Mays in the 1954 World Series (e.g., see http://en.wikipedia.org/wiki/The_Catch_(baseball)). He managed to catch a seemingly uncatchable ball, after looking at the ball and running to the interception location about 475 feet (145 m) from the home plate [1]. Willie Mays's catch made it to an illustration accompanying the contribution of Chodosh, Lifson, and Tabin in the 1995 volume of the journal *Science* [2]. These authors claimed that Willie Mays, and other adept outfielders, do not need to track the ball with their gaze because they are able to predict where and when to intercept the ball from the initial part of the ball trajectory. This will be the issue that we address in the present contribution: Does it suffice to view only the initial part of the ball's flight to predict the interception location or do fielders continuously track the ball with their gaze while running to that interception location?

Two types of strategy for the interception of moving targets have been distinguished in the literature (e.g., see [3]–[9]). On the one hand are the *predictive* strategies. In the context of fly-ball catching, this type of strategy amounts to looking at the ball's trajectory and predicting the interception location from the initial conditions of the ball's trajectory (i.e. its initial velocity and initial angle; cf. [10]). It should be noted that the use of such predictive strategy depends on a priori knowledge about gravity and air resistance. Because of drag and spin, fly balls do not follow parabolic trajectories (cf. [1], [8], [11]), which implies that a sophisticated internal model of ball-flight dynamics would have to be postulated.

An alternative to a predictive strategy is to use continuous visual guidance of locomotion on the basis of prospective information. Rather than having to know the interception location and time from early conditions, *prospective strategies* (e.g., see [12]–[14]) involve continuously available information that can be used to know whether the current action (e.g., running speed) will lead to a successful interception. In the context of the interception of fly balls, one such model states that if a fielder keeps the ball moving on a linear optical trajectory (LOT), he or she will arrive at the interception location in time, without knowing when and where the interception will take place [15]–[18]. The LOT strategy boils down to making sure that the horizontal and vertical components of the gaze angle (the angle between the heading and the gaze direction) change proportionally. Locomotion patterns of fielders running to catch fly balls travelling to locations in front or behind, and to the side of the fielders' initial positions have been reported to be in line with a LOT strategy (e.g., [16], [19]). However, several authors have claimed that keeping a linear optical trajectory is not sufficient to guarantee interception because linear optical trajectories can also occur for unsuccessful interceptions [20]–[23]. Furthermore, for balls approaching a fielder along the sagittal plane, a LOT strategy cannot be applied because there is only a vertical gaze angle; because there is no horizontal component of the gaze angle, all ball trajectories, whether leading to catches or not, will result in linear optical trajectories.

When a fly ball approaches along the sagittal plane, only running in the forward and backward direction needs to be controlled. In 1968, the physicist Neville Chapman [24] considered the mathematics of the situation of a fly ball on a parabolic trajectory approaching a fielder head on. He showed that the rate of change of the tangent of the gaze angle (the angle between the line of gaze and the horizontal, assuming that the gaze is directed at the ball) would remain constant if the fielder runs to the interception location at a constant speed. Thus, for fielders to arrive at the right place in the right time, the Chapman strategy amounts to keeping this rate of change constant. Because the rate of change of the tangent of the gaze angle is equivalent to the speed of the projection of the ball onto an image plane, and because keeping speed constant is equivalent to keeping acceleration at zero, the Chapman strategy is also known as the Optical Acceleration Cancellation (OAC) strategy (cf. [25], [26]; see also [6], [21], [27]).

Empirical studies have shown that participants, running to catch fly balls, show locomotion patterns that are consistent with the use of the OAC strategy [6], [26], [28], [29]. Because the OAC strategy is a strategy based on prospective information, it predicts that locomotion paths will differ for balls that land in the same spot but with different trajectories. This has been demonstrated in catching cricket balls [6], baseballs [23], and in heading virtual soccer balls [22].

The Chapman strategy specifically applies to fly balls that approach the fielder head on. As mentioned before, the textbook (e.g., [30], [31]) candidate complementary strategy to deal with the lateral component of running is the LOT strategy. Recent research using virtual reality has shown that the LOT strategy might not be the final answer [22], [23], and other strategies to complement the OAC strategy have been put forward (e.g., strategies of keeping constant the bearing angle—the CBA strategy, see [24], or its first temporal derivative—see [21]). If fielders control their locomotor trajectories on a moment-to-moment basis and use prospective information, they need to rely on a constant informational coupling with the approaching fly ball. However, according to Chodosh and colleagues [2] (cf. [1], [10], [11]), there is no need for such continuous visual coupling because fielders are capable of predicting the future landing location of the ball based on early available information of its trajectory: These authors argued that real fielders, like Willie Mays, simply look at the ball, predict the interception location, run there at maximal speed, and wait for the ball to arrive. Quite surprisingly, the issue of whether or not the catching of fly balls involves a constant visual coupling has not yet received much scientific attention. A notable exception is the study by Oudejans, Michaels, Bakker, and Davids [32], which examined gaze direction of fielders confronted with approaching fly balls.

Oudejans and colleagues [32] were interested in the potential contribution of extraretinal systems for picking up the information to guide running to intercept fly balls (see also [29], [33], [34]). They argued that if the ball is tracked with gaze, not only the retinal system but also vestibular or proprioceptive systems might be used to pick up optical acceleration. Participants were equipped with a gaze-tracking system, and were allowed to make a few steps in the right direction for fly balls projected at them head on. Because the gaze tracker was connected with a cable to the recording unit, participants could only move about one or two steps forward or backward. Interesting in the present context is the finding that participants in the Oudejans et al. study, indeed, continuously kept their eyes on the ball, by moving both their heads and eyes.

When using a predictive strategy, fielders obviously need to look at the ball during the initial part of its flight. Certainly, there is no need to keep the eyes on the ball during its entire flight. Although the use of a prospective strategy does not necessitate such continuous tracking of the ball (intermittent looking at the ball would suffice), the finding that participants do continuously track the ball would fit the use of a prospective strategy better than it would the use of a predictive strategy. The present paper reports an experiment in which we tracked the gaze of participants in a setting in which approaching fly balls either were within their locomotor reach (i.e., balls were catchable) or were not within their locomotor reach (i.e., balls were uncatchable). Importantly, the gaze tracker that we used was mobile, and allowed the participants to use their natural range of motion. That is to say, whereas Oudejans et al. [32] have shown that their participants continuously tracked the balls with their gaze for balls falling at or near the initial position of the participant or when simply watching balls that landed farther away than two steps, the present study allows to establish this behavior while participants are free to run much greater distances, even reaching their top speeds. Furthermore, we studied two situations. In line with the majority of previous studies on catching fly balls, we considered balls that would fly close enough to the participants that they would be able to arrive at the interception location in time. In addition, we also studied the situation in which balls were projected so far away from the participants' starting position that they would not be able to reach the ball before it would hit the floor. We instructed participants to indicate when they knew that a ball would be uncatchable, and when this occurred we inspected the direction of gaze up until this point. In short, the present study considered running to catch fly balls under the demanding circumstances as seen in regular ball games. Tracking the ball might be regarded much more difficult when running close to full speed. When even under these strenuous conditions we would find pursuit tracking of the ball, we argue, this gaze behavior must have a functional origin, which most probably would be related with continuous visual control.

## Methods

### Participants

Ten female volunteers (mean age 21.7±2.2 years) participated in the experiment. To be included, they needed to have at least two years of experience in ball sports. All participants reported normal, or corrected to normal (lenses) vision. Prior to the experiment, participants were informed about the procedure of the study and gave their written informed consent. The study was approved by the Ethics Board of the Center for Human Movement Sciences (University Medical Center Groningen, the Netherlands), and the protocol was in accordance with the Declaration of Helsinki.

### Apparatus

To determine the point of gaze (PoG), participants were equipped with a monocular, mobile eye tracker (Mobile Eye, Applied Science Laboratories, Bedford, MA). The tracking system consists of a scene camera (recording the field of view of the participant), and an optics module that consists of a near infrared light source and an eye camera. All components are mounted on a pair of lightweight spectacles. Calculation of the point of gaze is based on ‘dark pupil tracking’ and involves detection of the center of the pupil and the reflection of a cluster of three infrared LEDs on the cornea. Eye rotations are calculated from the angle and length of the vector connecting the pupil center and the corneal reflection. After calibration (see below), eye rotations are mapped onto the scene view, establishing the PoG in the scene. Interleaved images of the eye camera and the scene camera were recorded on tape using a portable video recorder (Sony GV-D1000E DVCR), at 30 Hz. The PoG was represented in the scene view by a crosshair with an approximate accuracy of 1° visual angle. The visual range of the eye tracker is 50 degrees horizontally, and 40 degrees vertically. The weight of the system, excluding the video recorder, is 76 grams. During testing, the video recorder was worn in a pouch around the waist, and allowed near-normal mobility for the participant.

The eye tracker was calibrated using a 3.3 m high by 4.4 m wide grid with 20 equally spaced points (4 rows of 5 dots each), representing a visual angle of 16.9° in the horizontal and 12.6° in the vertical direction. During calibration, the participants were positioned 15 meters from the grid, while their head rested on a chinrest that fixated the head. The gaze tracker was calibrated prior to the start of the experiment, after each set of 18 trials, and also when the participant indicated that the tracker had changed its position on the head during testing.

### Setup and procedure

The experiment was performed in a well-lit gymnasium (50×30×10 m). A ball-projecting machine (Louisville Slugger, type UPM45 Blue Flame) with adjustable force and projection angle was used to deliver tennis balls along the sagittal plane of the participant, at different projection distances. Because the projection angle could be manipulated only within a limited range, we used wooden blocks that were placed underneath the ball projection machine to generate the desired trajectories. The projected distance of the fly balls was varied systematically by adjusting the projection force and angle, and ranged approximately from 10 to 29 m. The apex of the trajectory was about 8.5 m for every trial, so that all fly balls had an approximate flight time of 2.5 s. The ball-projecting machine was occluded from sight to prevent visual anticipation of the ball's trajectory.

Participants completed 54 trials. The initial position of the participant was 20 m from ball projection, and was identical in all trials. At the start of each trial, the experimenter verbally cued the participant before ball delivery. Participants were instructed not to make a dive to perform a successful catch. Other than that, participants were free to move as they felt necessary to catch the ball. No instructions were given with regard to catching strategies (e.g. overhand or underhand catching). Not all projected balls were catchable. Participants were instructed to call ‘no’ at the instant that they realized that they were unable to catch the ball.

### Data analysis

We used EyeVision software (Applied Science Laboratories, Bedford, MA) to convert the video data that were stored on tape into AVI files. We analyzed the data from the moment of ball projection until the moment the ball was either intercepted or until the moment that the participant indicated that the ball was uncatchable by calling ‘no’. We used the audio signal from the internal microphone of the eye tracking system to detect these moments. More specifically, we marked the first video frame in which the sound of ball projection was audible as the first video frame for further analysis. The final frame that was analyzed for each trial corresponded either with the first frame in which the sound of the ball hitting the hand of the participant was audible, or the first frame in which the sound of the participant calling ‘no’ was audible. Audio analysis of the video data was performed in Adobe Premiere CS6.

To assess whether gaze tracked the ball, we considered the distance between the point of gaze (PoG) and the ball image, for each video frame. We used the EyeVision software to establish the 2D position of the PoG in the scene plane. Next, we used ASL Results Plus GM software (Applied Science Laboratories, Bedford, MA) to filter invalid values for the PoG. With custom-made software in MATLAB (Mathworks R2012b), we determined the 2D position of the ball in the scene plane, by hand. Finally, we computed the absolute distance in pixels between the PoG and the ball in the scene image.

We assigned points of gaze to one of two categories: either on the ball (‘tracking’) or not on the ball (‘other’). A PoG was considered to be on the ball whenever the absolute distance between the ball and the PoG in the scene plane was smaller than 75 pixels (corresponding to 6.25° visual angle). Although theoretically this criterion allows for changes in distance of 150 pixels between successive frames to be assigned to the ‘tracking’ category, it turned out that these changes were smaller than 25 pixels in 95.8% of the frames identified as ‘tracking’, and that in only 0.4% of the ‘tracking’ frames the change was greater than 75 pixels.

For each trial, the relative contributions of frames associated with ‘tracking’ and ‘other’ behavior will be expressed as a percentage of the total number of frames that had a valid PoG. Furthermore, we will present median distances between the PoG and the ball, as well as interquartile ranges.

## Results

The relation between the PoG and the ball could be established in 74.7% of all video frames. In the remaining frames, the relation between the ball and the PoG could not be assessed, either because the ball could not be identified in the video frame or because the PoG was lost. Unsuccessful calibration of the Mobile Eye led us to exclude the data from two participants from further analysis. Finally, 20 trials were excluded from further analysis because participants did not catch the ball but also did not indicate that it would be uncatchable (16 trials) or because we were unable to determine whether participants had touched the ball (4 trials).

Preliminary video analysis suggested that participants almost exclusively directed their point of gaze to the ball and rarely directed their gaze to locations elsewhere in the scene (for representative examples of a trial in which the ball was caught and of a trial in which a ball was judged to be uncatchable, see Figure S1 and Movies S1 and S2). The average median distance between the PoG and the ball was 24.26 pixels (with an average interquartile range of 22.67 pixels); medians (interquartile ranges) were 23.10 (20.28) pixels in the trials in which balls were caught (*n* = 230), and 25.85 (25.58) pixels in the trials in which balls were judged to be uncatchable (*n* = 168). Participants tracked the ball, on average, in 95.5% of the trial when they caught the ball, and in 92.9% of the trial when they called a ‘no’.

As detailed in the Methods section, video frames in which the distance between the ball and the PoG was more than 75 pixels formed the ‘other’ category. Further investigation of this category (representing 5.7% of all frames with a valid PoG) showed that 3.1% of all ‘other’ gaze behavior constituted meaningful gaze behavior and could be classified as ‘fixations’ (operationally defined as stable gaze for three or more consecutive frames). That is, fixations on items other than the ball accounted for 0.2% of all displayed gaze behavior.

**Figure 1:**
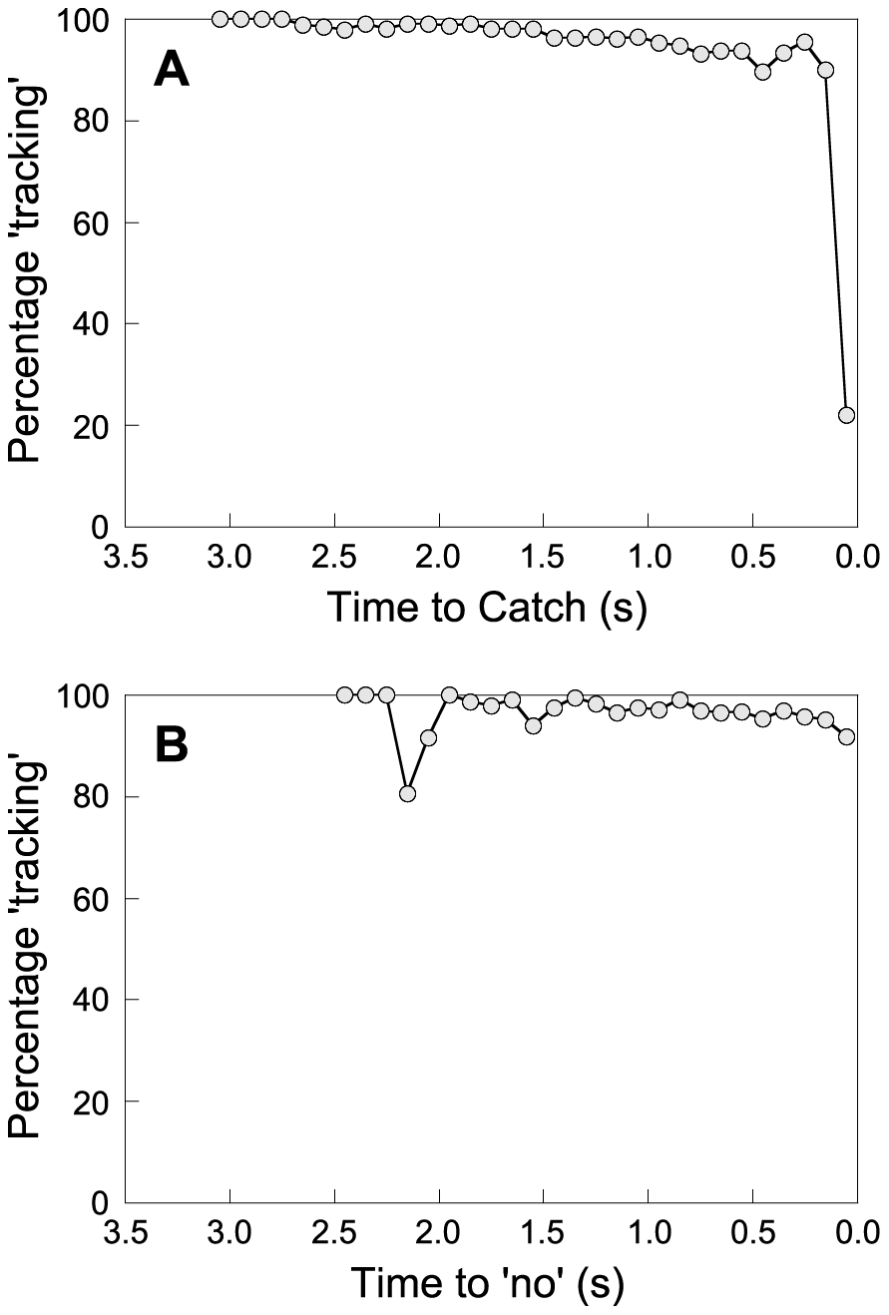
Percentage of tracking as a function of time. The number of frames in which participants tracked the ball expressed as a percentage of the total number of frames with valid data in a trial. A) Average percentages for trials in which the ball was caught; *t* = 0 represents the time of contact with the ball; B) Average percentages for trials in which the ball was judged to be uncatchable; *t* = 0 represents the time that a ‘no’ was called.

Figure 1 gives the percentage of frames that were categorized as ‘tracking’ as a function of time. Data points are represented in bins of 100 ms, combining sets of three consecutive video frames. In Figure 1A, which shows the trials in which the balls were caught, the abscissa represents the time until contact with the ball. It can be seen in Figure 1A that the contributions of tracking behavior to total gaze behavior remained relatively constant throughout the trial. Only the last 100 ms before the catch deviated substantially from this trend. Figure 1B represents the trials in which participants judged balls to be uncatchable. In Figure 1B, the abscissa represents the time until the moment that a ‘no’ was called. Also for this type of trials, the contribution of tracking behavior to total gaze behavior remained relatively constant throughout the trial. A slight deviation from this trend can be seen on the left side of Figure 1B (i.e., the two bins spanning from *t* = 2.2 to *t* = 2.0). Because these early bins included only few trials, the percentages of these bins were sensitive to the presence of single frames with a PoG that was coded as ‘other’. More particularly, one trial that had a few consecutive frames that were classified as ‘other’ early on in the trial (more than 2 s before the participant called ‘no’) was mostly responsible for the apparent decrease in tracking behavior.

## Discussion

In his famous catch in 1954, Willie Mays looked at the ball, turned his back to the ball while running, and finally looked back at the ball again. Is this the usual way for fielders running to catch a fly ball? Do they simply know where to run from a single glance on the ball's trajectory (cf. [1], [2], [10], [11]), or do fielders need continuous monitoring of the ball's position? The results of the present study show that participants running to catch an approaching fly ball continuously keep their eyes on the ball. Although the use of a predictive strategy would not preclude continuous tracking of the ball, and the use of a prospective strategy would not necessitate 100% tracking, the gaze behaviour of our participants suggests that their running is under continuous visual control, characteristic for a prospective strategy.

Earlier work by Oudejans et al. [32] showed that their participants reliably tracked the ball in both a fly-ball watching and a catching task. In the majority of trials administered by Oudejans and colleagues, participants were asked to simply observe fly balls approaching head on. Watching these balls resulted in pursuit tracking, with both head and eye movement contributing to keeping the gaze on the ball. In a catching condition, participants were allowed to move and actually catch the approaching balls. Because the gaze tracker used by Oudejans et al. was wired, it restricted the participants' mobility, such that they were only able to make a few steps to intercept a fly ball. As a consequence, balls in their catching condition had to be projected within a few meters from the participants' initial position. Also in the catching condition, participants tracked the ball with their gaze, although the contributions of head and eye movement to directing the gaze were different than in the watching conditions (see also [29]). The present study allowed participants to move as they would naturally do when catching a fly ball. With the mobile gaze tracker that we used, we were able to study gaze in situations comparable to real catching in the outfield.

Participants in the present study tracked the ball with their gaze nearly exclusively, regardless of the projected distance. Furthermore, they also showed tracking of balls that they realized were uncatchable. This latter condition is not commonly part of a study into the control of interception, although it is part and parcel of the reality of outfielders. The results suggest that the information for knowing that a ball cannot be caught should not be sought in a failure to keep tracking a ball. That is to say, our participants tracked the ball up until the moment that they indicated that the ball was out of their reach. They had no problems doing so, even when running at their maximum speed. Clearly, a failure to track the ball was no indication for the participants that an approaching fly ball would be uncatchable.

As discussed before, our results demonstrate that participants tracked the ball throughout its trajectory. We would like to stress that especially the fact that tracking continued to just before the actual catch speaks in favour of the use of continuous guidance rather than early prediction. Both the use of a predictive and of a prospective strategy would predict gaze pursuit during the early part of ball flight. However, when using a predictive strategy, in which the interception location and time are inferred from the first part of the ball trajectory, there seems to be no advantage of keeping an eye on the ball for the rest of its flight; continuous tracking fits more naturally with continuous visual control. Only during the very final part of the ball's approach, approximately during the final 100 ms, did tracking become inconsistent. A reason for this might be that the participants had actually stopped tracking the ball with their gaze because it was not needed for running to the interception location anymore. It has been suggested that fly ball interception consists of two phases; locomotion to the interception point and making the actual catch (e.g., see [23], [26]). The last 100 ms might reflect the latter phase. Alternatively, participants might have started to prepare for a follow-up action, such as throwing the ball to a teammate.

In conclusion, the present results paint a picture that is consistent with the use of a prospective strategy in dealing with the outfielder problem. Gaze data are not able to show indisputably that fielders do not use a predictive strategy, in which they know where to run from looking at the early part of ball flight. However, the finding that participants continuously kept their eye on the ball, while running several meters to catch a ball that might or might not be catchable, fits naturally with a continuous visual control on the basis of prospective information.

## Supporting Information

Figure S1 **Gaze in two representative trials.** Distance between the ball image and the point of gaze as a function of time. A) Gaze for a participant who successfully caught the projected fly ball (after 2.64 s); B) Gaze for a participant who indicated that the projected fly ball was uncatchable for her (after 1.37 s). See also Movies S1 and S2, which show scene camera recordings of these trials.
(PDF)

Movie S1 **Gaze in a trial in which the ball was caught.** Eye-tracker recording of a trial in which the participant caught the ball. The point of gaze is indicated by the red crosshair that overlays the scene-camera images. Figure S1A gives the distance between the ball and the point of gaze as a function of time for this trial.
(AVI)

Movie S2 **Gaze in a trial in which the ball was judged to be uncatchable.** Eye-tracker recording of a trial in which the participant judged the ball to be uncatchable. The point of gaze is indicated by the red crosshair that overlays the scene-camera images. Figure S1B gives the distance between the ball and the point of gaze as a function of time for this trial.
(AVI)
