# Supplementary material for: Keeping Your Eyes Continuously on the Ball While Running for Catchable and Uncatchable Fly Balls
Source: PLoS One. 2014 Mar 26;9(3):e92392. doi: 10.1371/journal.pone.0092392 (PMC3966785; doi:10.1371/journal.pone.0092392)
Supplement: Figure S1 — Gaze in two representative trials. Distance between the ball image and the point of gaze as a function of time. A) Gaze for a participant who successfully caught the projected fly ball (after 2.64 s); B) Gaze for a participant who indicated that the projected fly ball was uncatchable for her (after 1.37 s). See also Movies S1 and S2, which show scene camera recordings of these trials. (PDF) [file pone.0092392.s001.pdf]

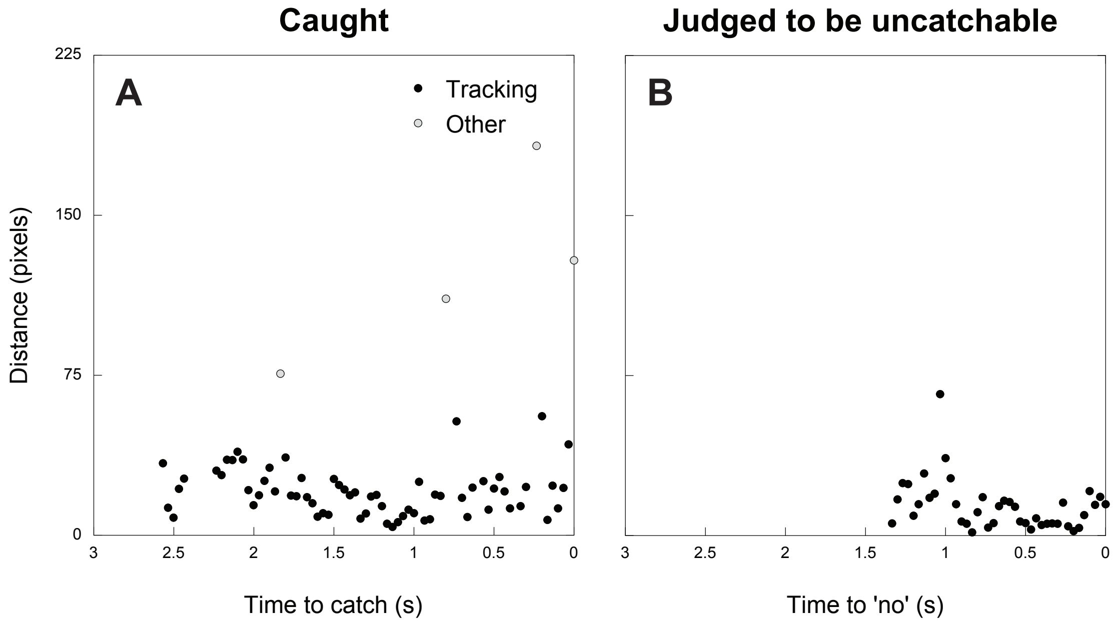

Figure S1. Gaze in two representative trials.

Distance between the ball image and the point of gaze as a function of time. A) Gaze for a participant who successfully caught the projected fly ball (after 2.64 s); B) Gaze for a participant who indicated that the projected fly ball was uncatchable for her (after 1.37 s). See also Movies S1 and S2, which show scene camera recordings of these trials.
